# Deciphering migraine pain mechanisms through electrophysiological insights of trigeminal ganglion neurons

**DOI:** 10.1038/s41598-023-41521-7

**Published:** 2023-09-02

**Authors:** S. Vongseenin, N. Ha-ji-a-sa, S. Thanprasertsuk, S. Bongsebandhu-phubhakdi

**Affiliations:** 1 Department of Physiology, Faculty of Medicine, Chulalongkorn University, Bangkok, 10330 Thailand; 2 Cognitive Clinical and Computational Neuroscience Center of Excellence, Chulalongkorn University, Bangkok, 10330 Thailand; 3 Present Address: Chula Neuroscience Center, King Chulalongkorn Memorial Hospital, Thai Red Cross Society, Bangkok, 10330 Thailand

**Keywords:** Neuroscience, Preclinical research, Neurology

## Abstract

Migraine is a complex neurological disorder that affects millions of people worldwide. Despite extensive research, the underlying mechanisms that drive migraine pain and related abnormal sensation symptoms, such as hyperalgesia, allodynia, hyperesthesia, and paresthesia, remain poorly understood. One of the proposed mechanisms is cortical spreading depression (CSD), which is believed to be involved in the regulation of trigeminovascular pathways by sensitizing the pain pathway. Another mechanism is serotonin depletion, which is implicated in many neurological disorders and has been shown to exacerbate CSD-evoked pain at the cortical level. However, the effects of CSD and serotonin depletion on trigeminal ganglion neurons, which play a critical role in pain signal transmission, have not been thoroughly studied. In this study, we aimed to investigate the association between CSD and serotonin depletion with peripheral sensitization processes in nociceptive small-to-medium (SM) and large (L) -sized trigeminal ganglion neurons at the electrophysiological level using rat models. We divided the rats into four groups: the control group, the CSD group, the serotonin depletion group, and the CSD/serotonin depletion group. We induced CSD by placing KCl on a burr hole and serotonin depletion by intraperitoneal injection of PCPA (para-chlorophenoxyacetic acid). We then isolated trigeminal ganglion neurons from all groups and classified them according to size. Using patch-clamp recording, we recorded the excitability parameters and action potential (AP) properties of the collected neurons. Our results showed that in SM-sized trigeminal ganglion neurons, the CSD-SM and CSD/serotonin depletion groups had a higher positive resting membrane potential (RMP) than the control-SM group (*p* = 0.001 and *p* = 0.002, respectively, post-hoc Tukey’s test). In addition, the gap between RMP and threshold in the CSD-SM group was significantly narrower than in the control-SM group (*p* = 0.043, post-hoc Tukey’s test). For L-sized neurons, we observed prolongation of the AP rising time, AP falling time, and AP duration in neurons affected by CSD (*p* < 0.05, pairwise comparison test). In conclusion, our study provides new insights into the underlying mechanisms of migraine pain and abnormal somatosensation. CSD and serotonin depletion promote the transmission of pain signals through the peripheral sensitization process of nociceptive small-to-medium-sized trigeminal ganglion neurons, as well as nociceptive and non-nociceptive large-sized trigeminal ganglion neurons.

## Introduction

Currently, various models are being used to better understand the pathophysiology of migraine headaches. Rats are commonly used for these models because their neuroanatomy and neurophysiology are similar to humans. Cortical spreading depression (CSD) has long been studied as the mechanism underlying the pathophysiology of migraine headaches. CSD plays a significant role in migraines, affecting both aura and pain. Functional imaging support the link between CSD and aura progression, with abnormal blood flow and heightened cortical activity observed in the occipital cortex during CSD-like waves^1^. CSD triggers vasodilation through neuro-inflammatory mediators, contributing to headaches^2^. Neuronal sensitization due to CSD is proposed as a crucial mechanism connecting aura and pain onset. Previous research has found that CSD can enhance nociceptive signals in many parts of the trigeminovascular system in rat models through a sensitization process. Peripheral trigeminovascular neuron sensitization results in the throbbing pain characteristic of migraines and can be aggravated by movements such as bending over, sudden head movements, coughing, and breath-holding^3^. Sensitization of higher-order trigeminovascular neurons results in extracephalic allodynia and hyperalgesia^4,5^. CSD also increases the firing rate, prolongs meningeal nociceptive neurons’ action potential, and accelerates the neuronal firing rate of central trigeminovascular neurons^6,7^. There are various ways to induce CSD in rat models, including placing KCl on or injecting it into the cerebral cortex, mechanical trauma, needle prick, and electrical triggers^8–13^. However, in vivo extradural application of KCl is the most neuroprotective method for rats^14^.

Serotonin depletion (5-HT depletion) is another mechanism known to exacerbate CSD-induced trigeminal nociception^15,16^. Clinically, normal levels of serotonin (5-HT) play an important role in regulating the neurocognitive system and preventing a variety of neurological disorders, including depression, autism spectrum disorder, dementia, Parkinson's disease, and migraines^17–25^. Evidence suggests that 5-HT depletion causes vasodilation, the important primary trigger of migrainous headaches^26^. In rat models, 5-HT depletion is induced by injecting para-chlorophenylalanine (PCPA) intraperitoneally. Electrophysiological studies have shown that 5-HT depletion increases the cortical excitability and sensitivity of the trigeminal nociceptive system by widening the duration of CSD waves at half amplitude, although it does not affect the average amplitude and duration of CSD. In addition, the frequency of the CSD wave also increases. The expression of c-Fos protein in the trigeminal nucleus caudalis (TNC) is also enhanced by 5-HT depletion^16^. Overall, many evidences suggest that 5-HT depletion plays a crucial role in migraine pathophysiology by enhancing CSD-induced trigeminal nociception. However, while the effects of CSD and 5-HT depletion on the sensitization of the cerebral cortex have been reported, their effects on first-order trigeminal ganglion neurons remain unknown.

The trigeminovascular pathway is considered the main pathway for the induction of migraine headaches. It consists of neurons with distinct properties, which contribute to different responses to CSD and 5-HT depletion. The first-order pseudounipolar neuron in the trigeminal ganglion receives input from the meninges and projects centrally to the spinal trigeminal nucleus, where the second-order trigeminovascular neurons relay ascending nociceptive information. The pseudounipolar neurons in the trigeminal ganglion can be classified into two sizes: small-to-medium (SM; ≤ 29 microns) and large (L; > 29 microns). These two categories differ in morphology, fiber innervation, protein expression, and electrophysiological properties. SM-sized neurons give rise to unmyelinated C-fibers and A-delta fibers, while L-sized neurons give rise to myelinated A-beta fibers. In terms of protein expression, CGRP is predominantly released by small unmyelinated C fibers, while CGRP receptors are expressed on small myelinated A-delta fibers. The interplay between these two small nociceptive fiber types assumes their pivotal role in nociceptive pathway at the dorsal horn^27–32^. Meanwhile, L-sized trigeminal ganglion neurons have been recently suggested by the electrophysiological study that they can function as both nociceptive and non-nociceptive neurons^33^. Additionally, regarding electrophysiological properties, the fibers of SM-sized neurons often respond to high-intensity mechanical, thermal, and chemical stimuli, while the fibers of L-sized neurons function as low-threshold mechanoreceptors^34,35^. Understanding the differences in the properties of these neurons may provide new insights into the pathophysiology of migraine and potential therapeutic targets.

In this study, our objective was to evaluate the peripheral sensitization induced by CSD and 5-HT depletion in nociceptive SM neurons and L-sized neurons using the patch-clamp recording. Our approach included measuring excitability parameters which were resting membrane potential (RMP), threshold potential and rheobase, and action potential parameters such as AP height, AP overshoot, AP rising time, and AP falling time. These parameters were compared among groups of rats with CSD, 5-HT depletion, and both CSD and 5-HT depletion. Furthermore, we analyzed the data separated into two subgroups based on neuron size (SM and L-sized), which determined their role as nociceptive or non-nociceptive trigeminal ganglion neurons. By examining the effects of CSD on SM and L-sized trigeminal ganglion neurons, we could demonstrate the peripheral sensitization process that is associated with migrainous pain and abnormal somatosensation. The findings of this study have the potential to uncover new insights into the pathogenesis of migraine and pave the way for novel treatment options.

## Methodology

### Experiment animal and cell groups

The study was carried out according to the Guide for the Care and Use of Laboratory Animals (8th Edition, National Academies Press). All methods were reported according to the ARRIVE guidelines. This study was conducted in accordance with ethical standards and received approval from the Animal Care and Use Committee of the Faculty of Medicine, Chulalongkorn University, Thailand (Approval No. 019/2561).

Adult male Wistar rats weighing 200–300 g were obtained from Nomura Siam International, Bangkok, Thailand and used in all experiments. The rats were housed in stainless steel cages placed in a well-ventilated room under a 12-h dark–light cycle. They had free access to food and water.

**Figure 1:**
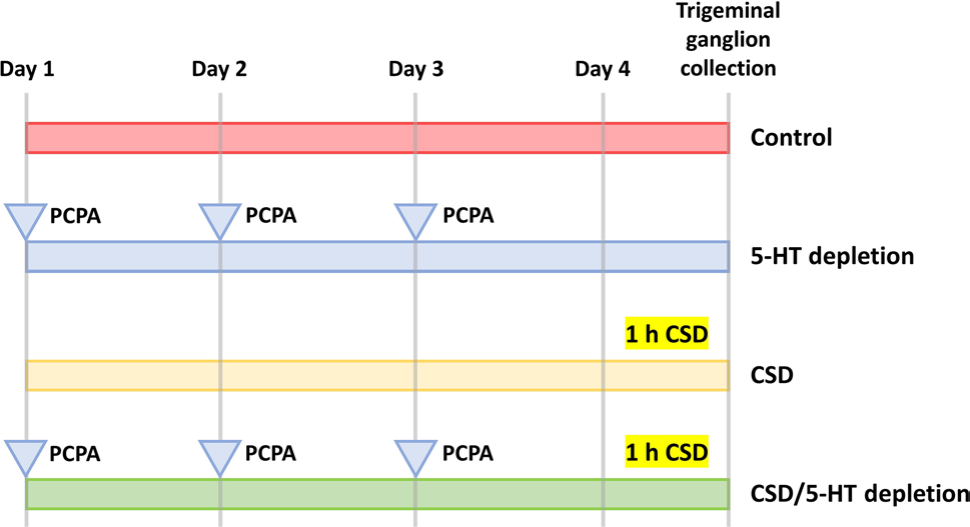
Experimental Timeline and Animal Grouping. A schematic representation of the experimental timeline and the grouping of animals in the study. Rats were categorized into four groups: control, serotonin (5-HT) depletion, cortical spreading depression (CSD), and combined CSD with 5-HT depletion (CSD/5-HT depletion). Control: Received intraperitoneal saline injections and NaCl placement on the burr hole as mimics. 5-HT Depletion: Intraperitoneal PCPA (100 mg/kg, 3 days) for serotonin depletion. CSD: Dura exposure, KCl (3 mg) applied for 1 h to induce CSD. CSD/5-HT depletion: PCPA injections (3 days), KCl applied after 24 h, mimicking CSD process.

Rats were divided into four groups: control, CSD, 5-HT depletion, and CSD/5-HT depletion. In the CSD group, the rats’ skulls were drilled until the dura mater was exposed, and KCl (3 mg) was placed on the burr hole for 1 h to induce CSD^36^. In the 5-HT depletion group, rats were injected intraperitoneally with PCPA (100 mg/kg body weight) for 3 days. In the CSD/5-HT depletion group, rats were first injected with PCPA (100 mg/kg body weight) intraperitoneally for three consecutive days. Within 24 h after the last dose of PCPA, KCl was placed in the burr hole by the same process as in the CSD group (Fig. 1). All rats in each group were euthanized by intraperitoneal injection of 70 mg/kg body weight of thiopental, administered slowly using a sterile syringe and needle. The injection site was carefully chosen to avoid any major blood vessels or organs, ensuring a controlled and humane administration of anesthesia for the experimental procedure. The trigeminal ganglion was then collected bilaterally and processed for primary culture. Subsequently, the trigeminal ganglia were sorted into eight subgroups based on neuronal diameter, consisting of small-to-medium-sized (SM; ≤ 29 microns) and large-sized (L; > 29 microns) neurons. The eight neuronal subgroups were the control-SM group, control-L group, CSD-SM group, CSD-L group, 5-HT depletion-SM group, 5-HT depletion-L group, CSD/5-HT depletion-SM group, and CSD/5-HT depletion-L group.

### Primary cell culture

The primary culture of trigeminal ganglion neurons was based on an improved protocol from the previous study^37^. Trigeminal ganglion neurons were placed in a dish filled with a mixture of Hank’s balanced salt solution (HBSS) and penicillin/streptomycin. After being washed twice in HBSS, the trigeminal ganglion neurons were cut into small pieces using a sterile razor blade in 1 mL of HBSS. The pieces were then transferred to a plastic tube and collagenase (100 μL; 2 mg/mL) and dispase (200 μL; 50 U/mL) enzymes were added. The samples were filtered through a 0.2 μm filter, incubated at 37 °C for 20 min, and centrifuged at 400 g RCF for 1 min. Papain was added and the samples were filtered again with a 0.2 μm filter, then incubated at 37 °C for 20 min. Subsequently, 2 mL of L-15 medium was added and the samples were centrifuged for 8 min at RCF 400 g. They were washed with 400 μL of F-12 complete medium and transferred onto a dish coated with laminin/PDL. The samples were then kept in the incubator at 37 °C and 5% CO_2_ for 3 h. Finally, the F-12 complete medium was changed twice and the samples were kept in the incubator for 18–24 h before being used for electrophysiological patch-clamp studies.

### Patch-clamp recording

To investigate the electrophysiological properties of trigeminal ganglion neurons, we performed whole-cell patch-clamp recordings using the same technique as in our previous studies. First, the trigeminal ganglion neuron samples were placed in plastic chambers and set on the microscope stage (Olympus BX51WI, Olympus, Japan). We then perfused the samples with an external solution at a flow rate of 1 mL/min and 25 °C. The external solution was composed of 145 mM NaCl, 5 mM KCl, 2 mM CaCl_2_, 1 mM MgCl_2_, 10 mM D-glucose, and 10% HEPES. We adjusted the pH to 7.40 with 1 M NaOH and the osmolality to 320 ± 5 mOsm/kg with d-glucose. Next, we measured the outer and inner diameters of the electrode, which were 1.5 mm and 0.86 mm, respectively (Sutter Instruments, Navato, CA, USA). We then filled the electrode with an internal solution containing 140 mM K-gluconate, 1 mM CaCl_2_, 10 mM EGTA, 10 mM HEPES, and 10 mM ATP. We adjusted the osmolality to 290 ± 5 mOsm/kg with d-glucose. To ensure successful patch-clamp recordings, we checked the tip resistances of the patch pipettes, which were between 6 and 8 MΩ, and performed Gigaseal. We also monitored the electrophysiological activity of each neuron during the patch-clamp recording. Only neurons with a resting membrane potential (RMP) more negative than − 40 mV, series resistance in the range of 10–25 MΩ, and capacitance less than 200 pF were included in our data analysis. We also noted the neuronal diameter (μm) and morphology. The membrane potentials of trigeminal ganglion neurons were manually set at − 60 mV and subjected to step pulse injections ranging from − 30 to 70 pA (in 10 pA increments) across 11 steps, each lasting 500 ms. We used the Axopatch 200B amplifier (Axon Instruments, CA, USA) and Clampex 10.2 software (Molecular Devices, CA, USA) to measure and record excitability parameters which are resting membrane potential (mV), threshold potential (mV), and rheobase (pA), and the action potential parameters including AP height (mV), AP overshoot (mV), AP rising time (msec) and AP falling time (msec). With these precise measurements and strict selection criteria, our patch-clamp recording technique ensured accurate and reliable data for our study of trigeminal ganglion neuron electrophysiology.

### Statistical analysis

The statistical analysis of the electrophysiological parameters followed a rigorous methodology. The mean and standard deviation (mean ± SD) were reported for all parameters. First, the normality of the data distribution was tested using the Shapiro–Wilk test. If the *p*-value of the test was less than 0.05, the data was considered not normally distributed and was subjected to the Kruskal–Wallis H test and pairwise comparison test. Conversely, if the *p*-value was higher than 0.05, the null hypothesis that the data was in normal distribution was accepted, and a one-way ANOVA test and post-hoc Tukey's comparison test were performed. IBM SPSS version 22, NY, USA was used for the analytical process, and the level of statistical significance was set at *p* < 0.05. By following this robust methodology, we ensured that the results obtained were reliable and statistically significant.

## Results

A total of 113 trigeminal ganglion neurons were included in the study and divided into two groups according to their size: small-to-medium (SM; ≤ 29 microns) -sized (n = 56) and large (L; > 29 microns) -sized (n = 57).

**Figure 2:**
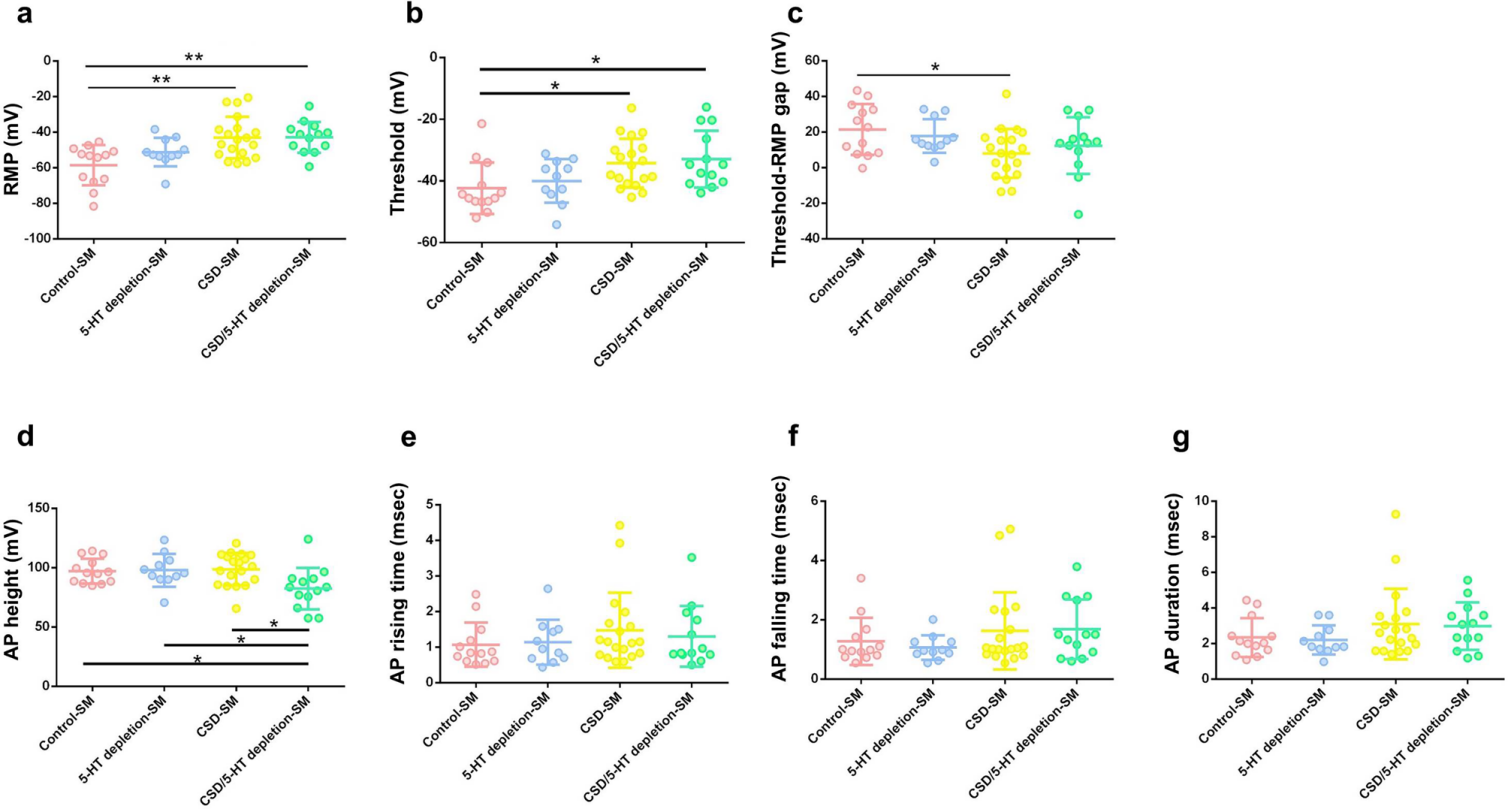
Excitability parameters and action potential parameters of small-to-medium (SM)-sized trigeminal ganglion neurons in the control-SM group, the 5-HT depletion-SM group, the CSD-SM group, and the CSD/5-HT depletion-SM group recorded by patch-clamp recording technique. (**a**) Resting membrane potential (RMP) (**b**) threshold potential (**c**) gap between RMP and threshold potential (threshold-RMP) (**d**) AP height (**e**) AP rising (**f**) AP falling (**g**) AP duration. The data shown represent mean ± SEM. Statistical differences between the control-SM group and other groups are displayed as *. The significance levels are indicated by *p < 0.05, **p < 0.01, ***p < 0.001 based on one-way ANOVA and post-hoc Tukey’s test.

In the SM-sized group, subjects were further classified into four groups: control-SM (n = 13), 5-HT depletion-SM (n = 11), CSD-SM (n = 19) and CSD/5-HT depletion-SM (n = 13). The mean diameter of SM neurons was 24.58 ± 2.53 μm, and there were no significant differences in size between the four groups (Table 1, *p* = 0.930, one-way ANOVA test), indicating that CSD and 5-HT depletion did not cause any morphological changes in the neurons. When Pearson’s chi-squared test was used to examine the bursting pattern of neurons among the four groups, no significant differences were found in the proportion of single-spike neurons and multiple-spike neurons (Table 1, *p* = 0.217, Pearson’s chi-squared test). A summary of the comparative electrophysiological parameters between the SM-sized groups is indicated in Supplementary Table 1. Regarding the excitability parameters, both the resting membrane potential (RMP) and the threshold showed significant differences between the experimental groups (Fig. 2a,b, *p* < 0.05, one-way ANOVA test). The post-hoc Tukey’s test revealed that the measured RMP of the CSD-SM (− 43.02 ± 11.67 mV) and CSD/5-HT depletion-SM (− 42.92 ± 8.74 mV) groups were significantly more positive than those of the control-SM (− 58.60 ± 11.30 mV) group (Fig. 1a, *p* = 0.001 and *p* = 0.002, respectively). Additionally, the threshold of the CSD-SM (− 34.24 ± 7.93 mV) and CSD/5-HT depletion-SM (− 32.94 ± 9.23 mV) groups were significantly higher than that of the control-SM group (− 42.39 ± 8.38 mV) (Fig. 2b, *p* = 0.038 and *p* = 0.024, respectively, post-hoc Tukey’s test). Furthermore, the differences between threshold and RMP were calculated and the CSD-SM group (7.99 ± 13.74 mV) had a significantly narrower gap (threshold-RMP) than the control-SM group (21.44 ± 14.36 mV) (Fig. 2c, *p* = 0.043, post-hoc Tukey’s test). In terms of action potential (AP) parameters, AP height was the only parameter that showed a significant difference between the groups (Fig. 2d, *p* < 0.05, one-way ANOVA test). The CSD/5-HT depletion-SM group had the lowest AP height (82.37 ± 17.53 mV), compared to the control-SM (97.12 ± 10.46 mV), CSD-SM (98.70 ± 13.66 mV), and 5-HT depletion-SM (97.75 ± 13.77 mV) groups (Fig. 2d, *p* = 0.047, *p* = 0.011, and *p* = 0.048, respectively, post-hoc Tukey’s test). However, other AP parameters among the experimental groups; AP rising time, AP falling time, and AP duration were not statistically significantly different (Fig. 2e,f,g).

**Table 1:** Demographic data of small-to-medium (SM)-sized trigeminal ganglion neurons in the control-SM group, 5-HT depletion-SM group, CSD-SM group, and CSD/5-HT depletion-SM group.

Parameters	Control-SM	5-HT depletion-SM	CSD-SM	CSD/5-HT depletion-SM	*p*-value^¶^
n	13	11	19	13	
Diameter (μm)	24.63 ± 2.88	24.80 ± 2.99	24.27 ± 2.29	24.79 ± 2.34	0.930
The number of single-spike/multiple-spike neurons	4/9	4/7	7/12	4/9	0.217

^¶^*p*-value ANOVA for diameter and *p*-value Pearson’s Chi-squared test for the number of single-spike/multiple-spike neurons.

**Figure 3:**
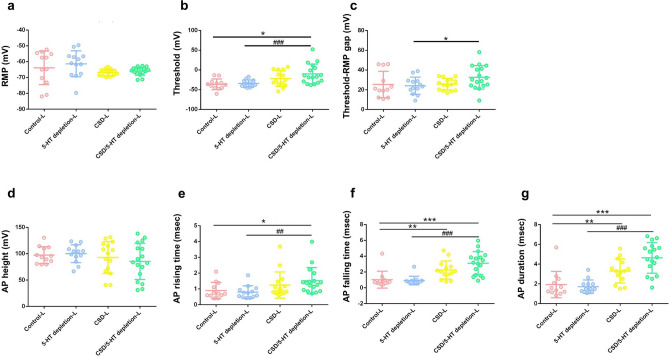
Excitability parameters and action potential parameters of large (L)-sized trigeminal ganglion neurons in the control-L group, the 5-HT depletion-L group, the CSD-L group, ﻿and the CSD/5-HT depletion-L group recorded by patch-clamp recording technique. (**a**) Resting membrane potential (RMP) (**b**) threshold potential (**c**) gap between RMP and threshold potential (threshold-RMP) (**d**) AP height (**e**) AP rising (**f**) AP falling (**g**) AP duration. The data shown represent mean ± SEM. Statistical differences between the control-L group and other groups are displayed as * while those between the 5-HT depletion-L group and the CSD/5-HT depletion group are displayed as #. The significance levels are indicated by *, ^#^*p* < 0.05, **, ^##^*p* < 0.01, ***, ^###^*p* < 0.001 based on Kruskal–Wallis and pairwise comparison test.

Similarly, in the group of L-sized subjects, four groups were identified, namely the control-L group (n = 12), the 5-HT depletion-L group (n = 13), the CSD-L group (n = 15), ﻿and the CSD/5-HT depletion-L group (n = 17). The average diameter of neurons in the L group was 33.96 ± 2.81 μm. In addition to the SM-sized neurons, no morphological changes were observed in the L-sized neurons after induction with CSD and 5-HT depletion, as confirmed by a one-way ANOVA test (Table 2, *p* = 0.133). The bursting profile of L-sized neurons was also unaffected, as evidenced by Pearson’s Chi-squared test, which showed no significant differences in the proportion of single-spike and multiple-spike neurons between the four groups (Table 2, *p* = 0.836). We summarized the data of comparative physiological parameters between the L-sized groups presented in Supplementary Table 2. Unlike the SM-sized group, the RMP values in the L-sized group were not statistically significant (Fig. 3a, *p* = 0.071, Kruskal–Wallis test). However, the threshold was found to be the most significant excitability parameter that differed between the four groups (Fig. 3b, *p* < 0.05, Kruskal–Wallis test). Pairwise comparison tests revealed that the CSD/5-HT depletion-L group (− 10.12 ± 25.00 mV) had a significantly higher threshold than both the control-L group (− 36.35 ± 13.39 mV) and the 5-HT depletion-L group (− 34.07 ± 8.94 mV) (Fig. 3b, *p* = 0.02 and *p* < 0.001, respectively). The gap between threshold and RMP was also calculated for this group, with the results indicating that the 5-HT depletion-L group (26.79 ± 13.15 mV) had a significantly narrower gap than the CSD-L group (55.62 ± 41.12 mV) and the CSD/5-HT depletion-L group (52.04 ± 26.81 mV) (Fig. 3c, *p* = 0.024 and *p* = 0.016, respectively, pairwise comparison test). In contrast to the comparison of AP properties between the SM-sized groups, AP height in the L-sized group was not significantly different (Fig. 3d, *p* = 0.695, Kruskal–Wallis test), while the AP rising time, AP falling time, and AP duration were the parameters that differed significantly between all groups (Fig. 3e,f,g, *p* < 0.05, Kruskal–Wallis test). The CSD/5-HT depletion-L group had significantly longer AP rising time, AP falling time, and AP duration (1.52 ± 0.83 mV, 3.12 ± 1.46 mV, and 4.64 ± 1.55 mV, respectively) compared to the control-L group (0.90 ± 0.51 mV, 1.03 ± 1.07 mV, and 1.93 ± 1.33 mV, respectively) (*p* = 0.017, *p* < 0.001, and *p* < 0.001, respectively, pairwise comparison test) and the 5-HT depletion-L group (0.80 ± 0.40 mV, 0.92 ± 0.54 mV, and 1.72 ± 0.68 mV, respectively) (*p* = 0.005, *p* < 0.001, and *p* < 0.001, respectively, pairwise comparison test) (Fig. 3e,f,g). Furthermore, the CSD-L group had a significantly prolonged AP falling time (2.76 ± 2.29 mV) and AP duration (4.00 ± 2.92 mV) compared to the control-L group (1.03 ± 1.07 mV) and the 5-HT depletion-L group (0.92 ± 0.54 mV) (Fig. 3f,g, *p* = 0.003 and *p* = 0.028, respectively, pairwise comparison test).

**Table 2:** Demographic data of large (L)-sized trigeminal ganglion neurons in the control-L group, 5-HT depletion-L group, CSD-L group, ﻿and CSD/5-HT depletion-L group.

Parameters	Control-L	5-HT depletion-L	CSD-L	CSD/5-HT depletion-L	*p*-value^¶^
n	12	13	15	17	
Diameter (μm)	33.77 ± 1.85	33.19 ± 2.24	33.82 ± 2.57	34.66 ± 2.22	0.570
The number of single-spike/multiple-spike neurons	10/2	12/1	14/1	15/2	0.836

^¶^*p*-value ANOVA for diameter and *p*-value Pearson’s Chi-squared test for the number of single-spike/multiple-spike neurons.

## Discussion and conclusions

Our experiments have revealed important findings that illustrate the association between cortical spreading depression (CSD) and peripheral sensitization, as well as the combined effect of CSD and serotonin depletion (5-HT depletion) on this phenomenon. We propose that the peripheral sensitization process is caused by changes in the excitability and action potential (AP) properties of trigeminal ganglion neurons, with the size of the neuron being a key factor that determines the pattern of neuronal response during this process. Our study shows that in small-to-medium (SM)-sized trigeminal ganglion neurons, there is an increase in excitability in the CSD-SM and CSD/5-HT depletion-SM groups compared to the control group. Furthermore, in large (L)-sized neurons, the AP parameters such as AP rising time, AP falling time, and AP duration were significantly prolonged in the CSD-induced neurons. These results can be attributed to various underlying mechanisms and further shed light on the association between CSD and CSD/5-HT depletion with clinical symptoms of migraineurs.

Previous research has shown the significant effects of CSD on neuronal excitability in various parts of the trigeminovascular system. CSD increases neuronal firing rate at both meningeal nociceptors and central trigeminovascular levels^6,7^. At the cortical level, 5-HT depletion has been shown to broaden the CSD wave at half amplitude and accelerate the generation of CSD waves^16^. Our study at the level of the first-order trigeminal ganglion neuron induced by CSD demonstrated no overall change in the neuronal bursting profile but significant changes in the electrophysical parameters of SM-sized nociceptive neurons, indicating hyperexcitability, consistent with other studies. Specifically, we observed a depolarization shift in the resting membrane potential (RMP) in the CSD-SM and CSD/5-HT depletion-SM groups and an elevation of threshold potential in the CSD-SM group. However, the narrowing of the gap between RMP and the threshold potential was observed in the CSD-SM group, suggesting that the more positive RMP caused a simultaneous rise in the threshold potential, but the depolarization shift of the RMP overcame the effect of the elevated threshold, leading to a more excitable state. The only AP parameter affected by CSD and 5-HT depletion in SM neurons was AP height, with the CSD/5-HT depletion-SM group exhibiting the lowest AP height among all groups. Elevated neuronal excitability and subsequent peripheral sensitization in SM-sized nociceptive neurons can lead to heightened expression of the inflammatory protein CGRP in the trigeminal ganglion. Additionally, this process can trigger the release of other ions and chemicals, including potassium, proton, and prostaglandin, further amplifying the peripheral sensitization^3,38^. CSD-induced NMDA receptor stimulation may also contribute to the peripheral sensitization process^39^. We hypothesize that the higher excitability of SM-sized nociceptive trigeminal ganglion neurons, along with the elevated level of protein expression and the adaptation of relevant receptors, contribute to migrainous aura and also an extreme sensitivity to pain (allodynia) in migraineurs during migrainous attack^40–43^. To conclude, it can be inferred that peripheral sensitization of SM-sized trigeminal ganglion neurons may potentially be the essential process connecting all migrainous symptoms including migrainous aura and migrainous pain.

In contrast, CSD and CSD with 5-HT depletion had varying effects on L-sized trigeminal ganglion neurons. In terms of excitability, the CSD/5-HT depletion-L group exhibited an increase in the threshold potential compared to the 5-HT depletion-L and control-L groups. Furthermore, there was a widening gap between RMP and threshold in the CSD/5-HT-L group compared to the 5-HT depletion-L group. Significant changes in the AP properties of this type of neuron were also observed. Specifically, the CSD/5-HT-L group demonstrated a prolongation of the AP rising time compared to the 5-HT depletion-L and control-L groups. Additionally, the AP falling time and AP duration were extended in the CSD-L group and the CSD/5-HT depletion-L group compared to the control-L and 5-HT depletion-L groups. In summary, in L-sized trigeminal ganglion neurons, CSD and CSD with 5-HT depletion resulted in the lengthening of the overall duration of the action potential, while decreasing the excitability of the neurons. Desensitization of L-sized neurons could potentially cause an imbalance between pain and somatosensory signal transmission at the trigeminal ganglion level. Based on current evidence, there are five types of L-sized trigeminal ganglion neurons, including type I, IIa, IIb, IIIa, and IIIb. These neurons respond differently to mechanical stimulation and sensory mediators such as serotonin, acetylcholine, and adenosine triphosphate. Although type IIIa and IIIb neurons are mostly insensitive to mechanical stimulation, they have the potential to function as nociceptive neurons, while I, IIa, and IIb neurons mainly function as non-nociceptive neurons. Moreover, for AP properties, the AP width of nociceptive type IIIa and IIIb was found to be significantly higher than in other groups when stimulated by depolarizing stimuli^33^. Considering the elongation of AP duration observed in L-sized trigeminal ganglion neurons due to CSD and CSD/5-HT depletion in our study, it is plausible to hypothesize that these factors might lead to an amplification of nociceptive signals conveyed by L-sized trigeminal ganglion neurons. Increased nociceptive signals, along with disproportionate pain and somatosensory signal, could potentially facilitate the transmission and peripheral sensitization of pain signals at the level of trigeminal ganglion neurons, leading to the aggravation of hyperalgesia and other somatosensory dysfunctions, including allodynia, hyperesthesia, and paresthesia^40,42,44^.

Our study has revealed that CSD and CSD/5-HT depletion contribute significantly to the peripheral sensitization process of both SM-sized and L-sized trigeminal ganglion neurons. This sensitization of peripheral neurons can further lead to hyperexcitability of central neurons or central sensitization, as has been demonstrated in several models^45^. Various mechanisms have been proposed to explain the underlying process of central sensitization. For example, inflammatory mediators such as CGRP and substance P released from primary afferent neurons have been observed to cause sensitization of second-order trigeminovascular dorsal horn neurons^46,47^. In addition, these mediators are believed to trigger the release of excitatory amino acids that activate NMDA receptors, resulting in dorsal horn sensitization^44^. Furthermore, elevated levels of c-Fos protein expression have been found in the TNC nucleus under the effect of CSD^48–50^. At the cortical level, CSD can also lead to upregulation of CGRP and pannexin 1 gene expression^51,52^. Notably, the expression of the c-Fos protein also increases in cortical neurons following both CSD and CSD/5-HT depletion^16^.

In conclusion, based on our study, CSD and CSD/5-HT depletion have been shown to play a crucial role in peripheral and central sensitization processes, which can result in various clinical symptoms from migrainous aura, migrainous headache and other migrainous pain-related symptoms. Specifically, up-regulation of inflammatory proteins and modulation of receptors induced by CSD and CSD/5-HT depletion can lead to sensitization of second- and third-order trigeminovascular nociceptive neurons. As a result, patients may experience cutaneous allodynia in the periorbital region when sensitization occurs in second-order neurons, and outside the referred pain area when it occurs in third-order neurons. These mechanisms are likely to be the main drivers of the clinical symptoms experienced by migraine patients, including hyperalgesia and abnormal somatosensation, such as allodynia, hyperesthesia, and paresthesia.

These findings can be advantageously applied at the clinical level. In the present day, the measurement of serum serotonin levels is readily available in numerous medical centers. This laboratory parameter holds the potential to predict the prognosis of migraine attacks in patients, encompassing the intensity of headaches, attack frequency, and individual susceptibility. In the forthcoming years, this parameter could form an integral part of a screening panel for identifying patients at a heightened risk of severe migrainous attacks. This proactive approach could facilitate early prevention strategies and swift migraine treatment interventions. Furthermore, considering the pivotal role of CSD in magnifying migraine pain, it is imperative to explore medications targeting channels and inflammatory mediators within the CSD pathway. This avenue of investigation holds promise for effectively curbing migrainous pain. While certain therapies related to calcitonin gene-related peptide (CGRP) have received approval for alleviating migraine pain, including CGRP receptor antagonists, CGRP monoclonal antibodies, and CGRP receptor^53^, further research is warranted in the domain of therapies focusing on different facets of the CSD pathway. For instance, despite the extended duration of its study, the efficacy and potential side effects of ketamine, a NMDA receptor antagonist, remain ambiguous^54,55^. Novel drugs like c-Fos inhibitors, currently employed primarily for pain relief in orthopedic conditions such as osteoarthritis and intervertebral disc pain^56^, may hold potential for application in migraine patients.

Our study has several limitations that should be considered. First, we only investigated the acute electrophysiological changes in neuronal cells. The long-term effects of CSD and serotonin depletion on neurons should be further investigated by examining changes in inflammatory protein expression through immunohistochemical studies. Additionally, pain perception was inferred from changes in electrophysiological parameters, rather than from direct observations of animals' behavior. Future studies may consider the use of inflammatory markers to more accurately determine pain perception in rat models. Furthermore, there is a possibility of protein degradation during the 18–24 h of incubation required before the study of patch-clamp recording of trigeminal ganglion neurons. This degradation could interfere with the accuracy of the model by affecting ion channel function or inflammatory processes. However, significant changes in excitability parameters and action potential properties were still observed in our study, indicating the validity of single-cell studies in assessing electrophysiological properties in this condition. In the future, in vivo patch-clamp recording may be used to overcome this limitation. Anywise, our approach enabled us to delve into the unique responses of SM-sized and L-sized trigeminal ganglion neurons to cortical spreading depression and serotonin depletion, yielding crucial insights into migraine pain mechanisms. Lastly, the patch-clamp recording was performed at the whole-cell level in our study. To disclose electrophysiological changes at a more detailed level, future studies should conduct patch-clamp recording at the single-ion channel level.

### Supplementary Information

Supplementary Table 1.

Supplementary Table 2.

## Data Availability

The datasets generated during and/or analysed during the current study are available from the corresponding author on reasonable request.
