# Supplementary material for: Deciphering migraine pain mechanisms through electrophysiological insights of trigeminal ganglion neurons
Source: Sci Rep. 2023 Sep 2;13:14449. doi: 10.1038/s41598-023-41521-7 (PMC10475091; doi:10.1038/s41598-023-41521-7)
Supplement: Supplementary file 1 — Supplementary Table 1. [file 41598_2023_41521_MOESM1_ESM.pdf]

Supplementary Table 1. Summary of the comparative electrophysiological parameters of ~~small to medium~~small-to-medium (SM)-sized trigeminal ganglion neurons in the control-SM group, ~~CSD-SM group,~~ 5-HT depletion-SM group, CSD-SM group, and CSD/5-HT depletion-SM group (p*-value < 0.05)**

Parameters	Control-SM	~~PCPA-SM~~5-HT depletion-SM	CSD-SM	~~CSD/PCPA-SM~~CSD/5-HT depletion-SM	*p*-value Shapiro-Wilk test	*p*-value ANOVA	*p*-value Kruskal-Wallis test
**RMP (mV)**	-58.60 ± 11.31	-51.25 ± 7.99	-43.02 ± 11.67	-42.92 ± 8.74	0.201	0.000*	-
**Threshold (mV)**	-42.39 ± 8.38	-40.02 ± 7.05	-34.24 ± 7.93	-32.94 ± 9.23	0.127	0.010*	-
**Threshold-RMP gap (mV)**	21.44 ± 14.36	17.71 ± 9.52	7.99 ± 13.74	12.32 ± 15.95	0.786	0.048*	-
**AP height (mV)**	97.12 ± 10.46	97.75 ± 13.77	98.71 ± 13.66	82.37 ± 17.53	0.334	0.010*	-
**AP rising (msec)**	1.06 ± 0.62	1.14 ± 0.63	1.47 ± 1.06	1.30 ± 0.86	< 0.001*	-	0.616
**AP falling (msec)**	1.27 ± 0.79	1.06 ± 0.41	1.62 ± 1.30	1.68 ± 1.00	< 0.001*	-	0.403
**AP duration (msec)**	2.34 ± 1.09	2.20 ± 0.83	3.10 ± 1.99	2.98 ± 1.33	< 0.001*	-	0.430
