# Supplementary material for: Deciphering migraine pain mechanisms through electrophysiological insights of trigeminal ganglion neurons
Source: Sci Rep. 2023 Sep 2;13:14449. doi: 10.1038/s41598-023-41521-7 (PMC10475091; doi:10.1038/s41598-023-41521-7)
Supplement: Supplementary file 2 — Supplementary Table 2. [file 41598_2023_41521_MOESM2_ESM.pdf]

Supplementary Table 2. Summary of the comparative electrophysiological parameters of ~~small to medium~~large (L)-sized trigeminal ganglion neurons in the control-L group, ~~CSD-L group,~~ 5-HT depletion-L group, CSD-L group, and CSD/5-HT depletion-L group (p*-value < 0.05)**

Parameters	Control-L	~~PCPA-L~~5-HT depletion-L	CSD-L	~~CSD/PCPA-L~~CSD/5-HT depletion-L	*p*-value Shapiro-Wilk test	*p*-value ANOVA	*p*-value Kruskal-Wallis test
**RMP (mV)**	-63.89 ± 10.59	-61.36 ± 8.21	-66.77 ± 2.06	-65.95 ± 2.56	< 0.001*	-	0.071
**Threshold (mV)**	-36.35 ± 13.39	-34.07 ± 8.94	-12.32 ± 41.90	-10.12 ± 25.00	< 0.001*	-	0.003*
**Threshold-RMP gap (mV)**	31.97 ± 19.61	26.79 ± 13.14	55.62 ± 41.12	52.04 ± 26.81	< 0.001*	-	0.004*
**AP height (mV)**	97.36 ± 16.11	100.12 ± 17.05	92.80 ± 30.29	85.48 ± 34.31	0.043*	-	0.695
**AP rising (msec)**	0.90 ± 0.51	0.80 ± 0.40	1.25 ± 0.83	1.52 ± 0.83	< 0.001*	-	0.002*
**AP falling (msec)**	1.03 ± 1.07	0.92 ± 0.54	2.76 ± 2.29	3.12 ± 1.46	< 0.001*	-	< 0.001*
**AP duration (msec)**	1.93 ± 1.33	1.72 ± 0.68	4.00 ± 2.92	4.64 ± 1.55	< 0.001*	-	< 0.001*
